# Development of Chinese food picture library for inducing food cravings

**DOI:** 10.3389/fpsyg.2023.1143831

**Published:** 2023-03-30

**Authors:** Hui-Ting Cai, Hong-Wei Zhang, Hui Zheng, Ting Xu, Lin Liu, Xu-Yan Ban, Jian-Zhong Di, Ti-Fei Yuan, Xiao-Dong Han

**Affiliations:** ^1^Shanghai Key Laboratory of Psychotic Disorders, Brain Health Institute, National Center for Mental Disorders, Shanghai Mental Health Center, Shanghai Jiao Tong University School of Medicine, Shanghai, China; ^2^Department of Metabolic and Bariatric Surgery, Shanghai Jiao Tong University Affiliated Sixth People’s Hospital, Shanghai, China; ^3^Co-innovation Center of Neuroregeneration, Nantong University, Nantong, China

**Keywords:** obesity, food craving, food addiction, salty foods, cue-induced craving

## Abstract

Cue-induced food cravings are strong desires directed toward specific foods, usually ones with high caloric content, and can lead to overeating. However, although food cravings vary according to individual preferences for specific high-calorie food subtypes, a structured library of food craving-inducing pictures including multiple categories of high-calorie foods does not yet exist. Here, we developed and validated a picture library of Chinese foods (PLCF) consisting of five subtypes of high-calorie foods (i.e., sweets, starches, salty foods, fatty foods, and sugary drinks) to allow for more nuanced future investigations in food craving research, particularly in Chinese cultural contexts. We collected 100 food images representing these five subtypes, with four food items per subtype depicted in five high-resolution photographs each. We recruited 241 individuals with overweight or obesity to rate the food pictures based on craving, familiarity, valence, and arousal dimensions. Of these participants, 213 reported the severity of problematic eating behaviors as a clinical characteristic. Under the condition of mixing multiple subtypes of high-calorie foods, we did not observe significant differences in craving ratings for high- and low-calorie food images (*p*_*tukey*_ > 0.05). Then, we compared each subtype of high-calorie food images to low-calorie ones, and found craving ratings were greater for the images of salty foods and sugary drinks (*ps* < 0.05). Furthermore, we conducted a subgroup analysis of individuals according to whether they did or did not meet the criteria for food addiction (FA) and found that greater cravings induced by the images of high-calorie food subtypes (i.e., salty foods and sugary drinks) only appeared in the subgroup that met the FA criteria. The results show that the PLCF is practical for investigating food cravings.

## Introduction

Food cravings are defined as a strong desire or urge to eat specific foods (Havermans, 2013). Many studies have consistently found that the intensity of cue-induced food cravings is positively associated with future eating and weight gain (for a meta-analysis, see Boswell and Kober, 2016). Cues indicating the availability of palatable foods can be seen everywhere in modern media-dominated society (Lesser et al., 2013; Powell et al., 2013) and continue to induce food cravings and drive overeating behaviors. Many studies have shown that the excessive response to food cues is an important factor contributing to the rapid rise in modern obesity rates (Morris et al., 2015; Chen et al., 2018; Pan et al., 2021), i.e., individuals with obesity or who may be prone to obesity may experience frequent and intense cue-induced food cravings and subsequent overeating behaviors (Stoeckel et al., 2008; Sun and Kober, 2020). However, although cue-induced cravings for high-calorie foods have been widely studied, previous studies have overlooked the distinctions between different categories of high-calorie foods. The majority of studies in the literature have simply compared cravings induced by mixed high-calorie food cues with those induced by low-calorie food or non-food cues (Hendrikse et al., 2015). Recent studies have suggested that cravings for different food types may be regulated by different neurobiological mechanisms (Gearhardt and Schulte, 2021) and influenced by different food cultures (Torrico et al., 2019). Thus, the distinctions between subtypes of high-calorie foods should not be ignored in further investigations of food cravings.

Although many food picture sets have been developed over the last decade (Foroni et al., 2013; Blechert et al., 2014; Charbonnier et al., 2016; Miccoli et al., 2016; King et al., 2018; Blechert, 2019; Toet et al., 2019), they all have shortcomings that limit their application to food craving studies in China: (1) lack of structured subtypes of high-calorie foods, (2) compatibility with Chinese food cultures, and (3) retention of natural scenes as a background to simulate exposure to food cues in daily life. For example, the latest updated picture set, Food-pics_extended (an updated version of Food-pics), collected 896 food images, enabling the evaluation of contrasts between high- vs. low-calorie foods, sweet vs. savory foods, and whole vs. processed foods (Blechert et al., 2014; Blechert, 2019). Its appropriateness is somewhat limited due to the standardized white background of the images and the collection of normative ratings primarily from German-speaking cultures. To our knowledge, only the Open Library of Affective Foods (OLAF), which includes 96 original images across four categories [low-calorie (fruits and vegetables) and high-calorie (savory and sweet)], presents foods within natural scenes to help maintain natural emotional responses (Miccoli et al., 2016). However, its food images and ratings are also based on Western culture. The CROss-CUltural Food Image Database (CROCUFID) recently collected food images and normative ratings from Western and Asian cuisines, but it only provides a rough classification of high-calorie foods (i.e., sweet vs. savory foods) (Toet et al., 2019). Therefore, there is still an unmet need for a structured set of high-calorie food pictures categorized into appropriate subtypes to investigate cue-induced cravings, which may help individuals with obesity or who may be prone to obesity cope with the overwhelming food cues of daily life.

Furthermore, obesity is a heterogeneous condition, and grouping all types of obesity together may lead to inconsistent findings about the phenotype of obesity because some clinical manifestations specific to certain subtypes may be masked or weakened (Field et al., 2013). For example, according to the incentive sensitization model of obesity (Berridge, 2009; Nijs and Franken, 2012), individuals with overweight or obesity are assumed to have greater cravings for high-calorie foods than for low-calorie foods, but recent studies have not shown consistent results in this regard (Hendrikse et al., 2015; Pawłowska and Kalka, 2015). Apart from the potential confounding effect of the distinctions between subtypes of high-calorie foods, another possible reason for the inconsistency could be these studies’ attempts to draw generalizations across subtypes of obesity, i.e., mixing eating-related characteristics in individuals with overweight or obesity. On the one hand, obesity and eating disorders (ED) are commonly comorbid conditions (Ivezaj et al., 2016), and researchers have found that individuals with comorbid obesity and ED experience more cue-induced food cravings than obese individuals without ED (Reents and Pedersen, 2021). That means the manifestation of food cravings may largely depend on the subtype distribution in a given sample, if the study fails to consider the heterogeneity in eating-related characteristics among the included individuals. On the other hand, since cravings are considered a core symptom of addiction (Sayette, 2016), a particular type of eating behavior known as “food addiction” (FA) might also significantly impact the manifestation of food cravings among individuals with overweight or obesity.

Food addiction is commonly operationalized by the Yale Food Addiction Scale (YFAS) (Gearhardt et al., 2016), which was adapted from the Diagnostic and Statistical Manual of Mental Disorders (DSM) criteria for substance use disorders to describe the addiction-like responses to certain foods. Measured by the YFAS, many individuals with overweight or obesity report symptoms of FA (Ivezaj et al., 2016), and the experience of FA symptoms might affect their behavioral and neural responses to cue-induced cravings. Neuroimaging studies have revealed that individuals who have obesity and meet FA criteria show increased activation in the superior frontal gyrus for highly processed food cues compared to individuals who have obesity but do not meet FA criteria (Schulte et al., 2019). Increased activation in the superior frontal gyrus is consistent with the neural response associated with cue-induced cravings in substance use disorders. Furthermore, integrated evidence suggests that obesity which meets FA criteria and substance use disorders share common neurobiological mechanisms, e.g., the dopamine system in the mesolimbic pathway is one of their common neurobiological substrates (for literature reviews, see: Volkow et al., 2013; Gupta et al., 2020). In individuals susceptible to FA, repeatedly consuming rewarding foods might provoke neural adaptations that result in reduced dopamine signaling in the reward circuitry, which might contribute to increased cravings and over-consumption (Volkow et al., 2006; Stoeckel et al., 2008). In addition, the addiction-like responses to different foods may be regulated by different neurobiological mechanisms. For example, the salted food addiction hypothesis posits that salted foods may act as mild opiate agonists, which can indirectly regulate dopamine signaling by reducing the activity of inhibitory interneurons rather than by activating dopaminergic neurons directly (Le Merrer et al., 2009; Gearhardt and Schulte, 2021).

Therefore, we seek to further investigate the manifestation of food cravings and resolve the inconsistency currently present in the literature regarding whether high-calorie foods evoke greater cravings in individuals with overweight or obesity by developing a structured picture library that can be used as a more sophisticated tool for measuring eating-related characteristics. Taking the above into account, we sought to develop a structured set of pictures of high-calorie foods, which we call the Picture Library of Chinese Foods (PLCF), based on the standardized International Affective Picture System (IAPS) procedure (Bradley and Lang, 2007). We designed the PLCF to include five subtypes of high-calorie foods, namely, sweets, starches, salty foods, fatty foods, and sugary drinks, to cover the subtypes of foods for which cravings frequently occur according to YFAS 2.0 (Gearhardt et al., 2016). Study participants were then asked to rate each picture in terms of familiarity, valance, arousal, and craving dimensions. The original pictures and associated ratings are available on the Open Science Framework website.^1^ Concerning the inconsistency of results finding greater cravings for high-calorie foods among people with overweight or obesity, we hypothesized that (1) the greater cravings for high-calorie foods occur only in certain subtypes of high-calorie foods rather than all high-calorie foods in general; and (2) the greater cravings for high-calorie foods are driven by individuals who meet FA criteria.

## Materials and methods

### Participants

From April 2021 to October 2022, we recruited 254 participants with overweight or obesity, defined as body mass index [BMI, equal to (weight in kilograms divided by height in meters)^2^]>24 from a Weight Loss Metabolism Clinic in China. All participants (1) were between 18 and 60 years old; (2) could read and understand the description of each item in the questionnaire; and (3) participated in the study voluntarily and agreed to fill out the online questionnaire carefully. We excluded 13 participants due to indicators that their responses may have been low quality, including outlier response times, incorrect answers to forced questions, low ratings in self-reported effort (rating <6), and abnormal responses to basic informational questions (for example, responses indicating fasting for more than 48 h) (Curran, 2016). After quality controls, our formal analysis included 241 participants, 213 of whom had completed the questionnaires measuring clinical characteristics. Using the YFAS 2.0, we divided the participants into two groups: those who did and those who did not meet FA criteria. More details are summarized in Table 1. This study was approved by the Ethics Committee of Shanghai Sixth People’s Hospital [2020-219-(1)]. All procedures followed the Declaration of Helsinki.

**TABLE 1:** Demographic and clinical characteristics of the raters.

Characteristics	Total sample	Individuals who did meet FA criteria	Individuals who did not meet FA criteria	*p*-value
	**Mean (SD)/*N* (%)**	**Mean (SD)/*N* (%)**	**Mean (SD)/*N* (%)**	
*N*	241	122	91	
Sex				
Female (%)	183 (84.7)	96 (82.8)	79 (88.8)	0.314
Age (year)	32.39 (6.99)	31.59 (6.87)	33.51 (6.75)	0.047
Education (year)	14.07 (2.62)	13.98 (2.64)	14.24 (2.65)	0.497
BMI (kg/m^2^)	38.86 (12.79)	39.10 (13.43)	38.63 (12.58)	0.797
**Baseline states**				
Fasting (hours)	5.70 (5.82)	5.43 (5.88)	5.50 (5.41)	0.932
Hungry (0–10)	3.30 (2.64)	3.21 (2.62)	3.21 (2.50)	0.990
Thirsty (0–10)	4.39 (2.67)	4.55 (2.73)	4.13 (2.61)	0.262
Craving (0–10)	4.13 (2.86)	4.35 (2.97)	3.77 (2.76)	0.146
Emotion (0–10)	5.66 (2.50)	5.07 (2.55)	6.20 (2.37)	0.001
**Food addiction (YFAS 2.0)**				
The average number of symptoms met the criteria	6.02 (3.64)^a^	8.05 (2.83)	3.31 (2.71)	<0.001
Frequency of cravings experienced in life	3.22 (2.31)^a^	4.13 (2.42)	2.00 (1.45)	<0.001
**Eating behaviors (EDE-Q)**				
The average value of the global score	4.16 (0.96)^a^	4.59 (0.84)	3.58 (0.81)^a^	<0.001

YFAS, Yale Food Addiction Scale 2.0; EDE-Q, eating disorder examination questionnaire.

^a^The values reported here are calculated from data of 213 participants who had completed the YFAS 2.0 and the EDE-Q.

### Materials

**FIGURE 1:**
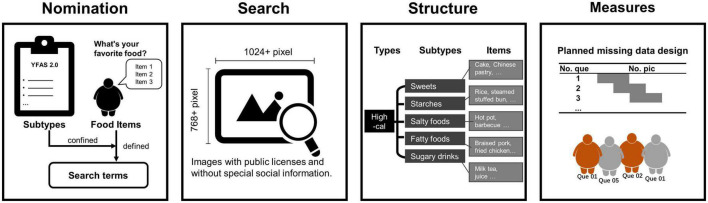
The development process of the Picture Library of Chinese Foods (PLCF). From left to right, this figure describes the steps to construct the PLCF. Step 1, nomination: we first defined subtypes of foods with reference to the YFAS 2.0, invited local people with obesity to nominate their favorite foods, and finally defined image search terms based on foods that were frequently nominated and fell into the predefined subtypes. Step 2, search: we searched the Internet for and downloaded publicly licensed images of the most frequently nominated foods. The inclusion criteria were: (1) resolution greater than 1,024 × 768 pixels; (2) the food was presented in the center of the image within a natural scene; and (3) no special social information, such as human faces or hands, appeared. Step 3, structure: the classification hierarchy of the PLCF was finalized. The picture library contains five food subtypes, each with four items depicted in five different images. Step 4, measures: the picture set was divided into ten subsets according to a planned missing data design. Each subset included twenty high-calorie images without food item repetitions and one low-calorie and one spoiled food image as the internal references. Each rater rated only one subset. More details are described in the section “Materials and methods”.

Figure 1 shows the development of the PLCF. We first defined the subtypes of high-calorie foods according to the YFAS 2.0 instructions. To ensure that the selected food items could effectively induce the local participants, before starting the food image search, we invited 31 individuals with overweight or obesity from the same Weight Loss Metabolism Clinic to nominate their three favorite foods through anonymous messages. There was no overlap between the 31 individuals involved in the food nominations and the participants in the picture rating sessions. We then searched for the food items that were nominated frequently and met the subtype requirements. Then, we downloaded images of these foods from image-sharing sites with public licenses. The image collection criteria were (1) resolution greater than 1,024 × 768 pixels; (2) the food was presented in its entirety and centered within a natural scene; and (3) no special social information, such as human faces or hands, appeared. In this manner we constructed a set of high-calorie food pictures for each of the five subtypes, with each subtype including four food items represented by five different images. In total, we selected 100 high-calorie food images from the Internet. We also collected five images of low-calorie foods and five of spoiled foods to provide an internal reference. Finally, we post-processed all images using Adobe Photoshop CC 2022, uniformly resized them to 1,024 × 768 pixels, and exported them in Joint Photographic Experts Group (JPEG) format.

### Measures

The YFAS 2.0 is a 35-item self-report measure that operationalizes indicators of FA (Gearhardt et al., 2016). It contains eleven symptom criteria and a clinical impairment criterion adapted from the Substance-Related and Addictive Disorders definition in the fifth revision of the DSM. Each item falls under one symptom criterion. All items on the YFAS 2.0 are continuous and have eight frequency response options that range from “Never” to “Every Day.” Different thresholds then dichotomize items to determine the symptom criterion by summing up the items under each criterion. A sum of item scores ≥1 indicates meeting the symptom criterion. The number of symptoms meeting the criteria indicates the severity of FA symptoms, ranging from 0 to 11. To meet the criteria of FA, an individual must meet both the clinical impairment criterion and at least two symptom criteria. The internal consistency coefficient in the current study was 0.97, indicating excellent consistency. Item 30 (“*I had such intense cravings for certain foods that I felt like I had to eat them right away.*”), measuring the frequency of cravings experiences in life, was chosen as an external criterion to validate the cravings induced by the PLCF.

The Eating Disorder Examination Questionnaire (EDE-Q) is a 28-item self-reported questionnaire designed to assess the range and severity of features associated with a diagnosis of eating disorder (ED) (Reas et al., 2006). It contains four subscales (Restraint, Eating Concern, Weight Concern, and Shape Concern) and a global score. The items in the four subscales have seven frequency response options ranging from “No Days/Not at all” to “Every Day/Markedly.” The mean of the four subscales is the global score; a higher global score indicates more problematic eating behaviors. The internal consistency coefficient in the current study was 0.86, indicating good consistency.

### Procedure

We followed a planned missing data design to conduct the image rating session (Bradley and Lang, 2007; Gu et al., 2021). First, we divided the total image set into ten subsets. Each subset included twenty high-calorie images without food item repetitions, one low-calorie image, and one spoiled food image. Then, we randomized the subsets across rating sessions, and the image display order was also randomized within each subset to avoid any sequential effect. We only asked each participant to rate one subset to avoid potential fatigue effects. Participants performed the rating procedure in a Chinese Weight Loss Metabolism Clinic through an online platform.^2^

Individuals with overweight or obesity could participate in rating sessions only after they had given informed consent. Participants reported basic demographic information (gender, age, years of education), BMI, and baseline status (hunger, thirst, desire to eat, mood) on an 11-point Likert scale. Then, they saw standardized rating instructions and were asked to evaluate each food image in terms of familiarity, craving, valence, and arousal from 0 (“Not at all” or “Negative” in valence) to 10 (“Very Much” or “Positive” in valence) separately. Finally, they completed the EDE-Q and the YFAS 2.0 to help investigate the relationship between the severity of problematic eating behaviors and the food cravings induced by high-calorie food images. Each rating session was performed consecutively within a single session.

### Statistical analysis

We processed all data using R (version 4.1.2)^3^ and jamovi (version 2.2.5).^4^ We reported continuous variables (such as the Likert scale with normal distribution) as the mean±standard deviation (*SD*) and reported non-normal variables as the median and range. We first checked for any outlier pictures with any rating dimension exceeding the mean ± 3 *SD*, which would be removed from further analyses. Then, we conducted reliability and validity analyses to examine the measurement characteristics of the PLCF. Reliability analysis refers to the internal consistency coefficient (Cronbach’s alpha, α), with 0.8 < α < 0.9 indicating good consistency and α > 0.9 indicating excellent consistency (Henson, 2001). The validity analysis included internal validity (whether high-calorie foods were superior to other food images) and criterion validity (correlation between cravings induced by high-calorie food images and life craving experience frequency). The criterion was the frequency of craving experiences in life measured by a continuous item in YFAS 2.0 (“*I had such intense cravings for certain foods that I felt like I had to eat them right away.*”). After confirming that the measurement characteristics of the PLCF were good enough, we further explored the potential influencing factors of cue-induced cravings by repeated-measures analysis of variance (ANOVA) and Pearson correlation analysis. For all analyses, we set the significance level to 0.05, performed *post hoc* pairwise comparisons for ANOVA using the Tukey correction, and performed multiple comparison corrections for correlation analysis using the False Discovery Rate (FDR) correction (Benjamini and Hochberg, 1995).

## Results

### Reliability analysis

All images survived the outlier check and were therefore included in subsequent analyses. We evaluated the internal consistency with Cronbach’s alpha reliability coefficients and calculated each rating dimension within 20 high-calorie items as 0.949 (95% confidence interval (CI) = [0.933, 0.959]) for familiarity, 0.895 (95% CI = [0.869, 0.913]) for craving, 0.883 (95% CI = [0.851, 0.907]) for valence and 0.899 (95% CI = [0.874, 0.919]) for arousal ratings. All of the dimensions showed good reliability.

### Validity analysis

**FIGURE 2:**
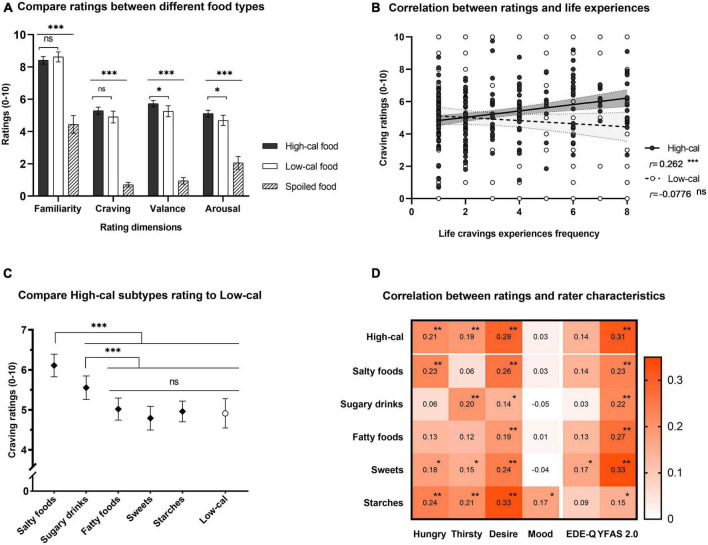
Statistical analysis of PLCF ratings. **(A)** Histogram of one-way repeated-measures ANOVA results for each rating dimension. All ratings of palatable food (i.e., high- and low-calorie) images were significantly higher than those of spoiled food. Ratings of high- and low-calorie images significantly differed on the valence and arousal dimensions but not on the craving dimension. Solid bars indicate high-calorie foods, hollow bars indicate low-calorie foods, and diagonal lines indicate spoiled foods [same in panels **(A–C)**]. **(B)** Scatter chart of the Pearson correlation between craving ratings and craving experience frequency in daily life. The frequency of craving experiences in life was positively correlated with the cravings induced by high-calorie images and was uncorrelated with those induced by low-calorie images. A linear fit was conducted separately, and the shading within the dotted lines indicates the 95% CI. **(C)** Dot plot of the results from one-way repeated-measures ANOVA with six levels (i.e., five subtypes of high-calorie food and low-calorie food). The cravings induced by salty food and sugary drink images were greater than those induced by low-calorie images. The diamond symbols indicate subtypes; the circles indicate food types. **(D)** Heatmap of the correlation coefficient between the craving ratings of high-calorie food images and rater characteristics. The cravings induced by high-calorie food images were positively correlated with the baseline desire to eat and the severity of food addiction measured by YFAS 2.0. However, there was only one correlation with the severity of eating disorder symptoms measured by EDE-Q. EDE-Q, eating disorder examination questionnaire; YFAS, Yale Food Addiction Scale 2.0. For all subplots, *** indicates *p* < 0.001, ** indicates *p* < 0.01, * indicates *p* < 0.05, and “ns” indicates no significance. The error bar indicates the 95% CI.

To test the internal validity of the high-calorie images, we subjected each rating dimension to one-way repeated-measures ANOVA with food type (high-calorie, low-calorie, and spoiled food) as a within-subject factor. The main effect of food type was significant in all ANOVA tests [all *ps* < 0.001; for familiarity, *F*(2,480) = 221, *partial* η^2^ (η^2^_*p*_) = 0.479; for craving, *F*(2,480) = 422, η^2^_*p*_ = 0.637; for valence, *F*(2,480) = 430, η^2^_*p*_ = 0.642; for arousal, *F*(2,480) = 114, η^2^_*p*_ = 0.322]. The results of the *post hoc* tests with Tukey corrections showed that the palatable food (both high- and low-calorie) images induced substantially greater cravings than the spoiled food images [for high-calorie, mean difference (*MD*) = 4.582, standard error (*SE*) = 0.124, *t*(240) = 36.92; for low-calorie, *MD* = 4.195, *SE* = 0.193, *t*(240) = 21.71; all *p*_*tukey*_ < 0.001, Figure 2A], and the differences were also significant in terms of the familiarity, valance, and arousal ratings. When comparing palatable food images, there was no significant difference in familiarity [*MD* = −0.208, *SE* = 0.125, *t*(240) = −1.66, *p*_*tukey*_ = 0.224] and craving ratings [*MD* = 0.387, *SE* = 0.198, *t*(240) = 1.96, *p_*tukey*_* = 0.126] between low- and high-calorie foods. However, the valence [*MD* = 0.475, *SE* = 0.184, *t*(240) = −2.59, *p*_*tukey*_ = 0.028] and arousal ratings [*MD* = 0.430, *SE* = 0.173, *t*(240) = 2.49, *p*_*tukey*_ = 0.036] were significantly higher for high-calorie images than for low-calorie images, supporting that the two types of images are distinguishable. We further examined the criterion validity to verify the distinction between the two types of palatable food images. The Pearson correlation analysis showed that the frequency of craving experiences only correlated with cravings induced by the high-calorie images [*r*(213) = 0.262, *p* < 0.001], not with cravings induced by low-calorie images [*r*(213) = −0.078, *p* = 0.260, Figure 2B]. These results suggested that the PLCF was an effective tool for measuring high-calorie food cravings in the population with overweight or obesity.

### The heterogeneity of high-calorie food subtypes

To test the impact of the high-calorie food subtypes on cue-induced cravings, we adopted a one-way repeated-measures ANOVA with five subtypes of high-calorie and low-calorie food images as repeated-measures levels. We summarized the results in Figure 2C. The main effect of the subtypes was significant [*F*(5,1200) = 17.9, *p* < 0.001, η^2^_*p*_ = 0.069], and *post hoc* analysis revealed that both salty foods [*MD* = 1.209, *SE* = 0.216, *t*(240) = 5.585, *p*_*tukey*_ < 0.001] and sugary drinks [*MD* = 0.655, *SE* = 0.224, *t*(240) = 2.918, *p*_*tukey*_ = 0.044] induced greater cravings than low-calorie food images. The cravings induced by other subtypes of high-calorie images did not significantly differ from those of low-calorie images (all *p*_*tukey*_ > 0.05). These results suggested that the absence of differences between cravings induced by high- and low-calorie food images resulted from the heterogeneity of high-calorie food subtypes.

To further explore the relationship between craving ratings and individual characteristics, we performed a Pearson correlation analysis between craving ratings and rater characteristics for all high-calorie food images and the five subtypes. We summarized the significance of all coefficients defined by the FDR corrected *p*-values in Figure 2D. We observed that the cravings induced by high-calorie food images positively correlated with the baseline desire to eat [the range of *r*(213) was (0.14, 0.33) and significant across all subtypes, all *p*_*fdr*_ < 0.05] and with FA severity measured by YFAS 2.0 [the range of *r* (213) was (0.15, 0.33) and significant across all subtypes]. In contrast, there was a weak correlation with the severity of ED symptoms measured by EDE-Q [only significant with images of sweets, *r*(213) = 0.17, *p_*fdr*_* = 0.029]. These results suggested that the high-calorie cravings among individuals with overweight or obesity might also depend on their heterogeneity in eating-related characteristics, especially the severity of FA symptoms.

### The heterogeneity of populations

**FIGURE 3:**
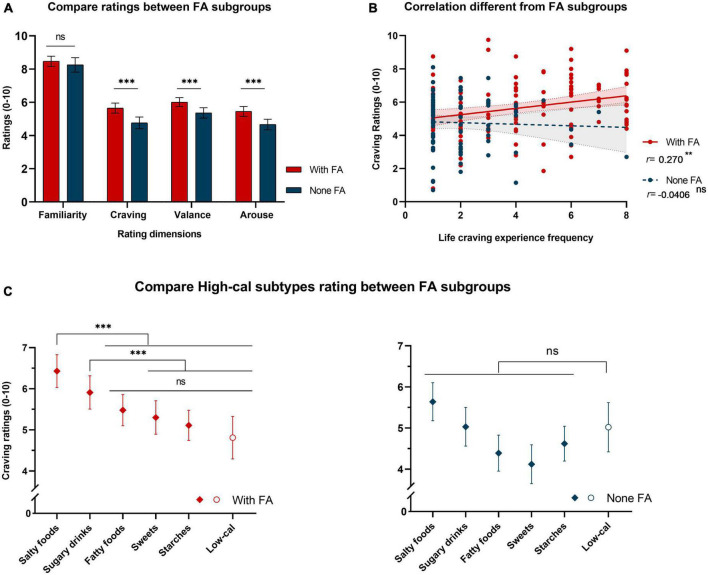
Statistical analysis of PLCF data stratified by FA criteria. **(A)** Histogram of independent samples *T*-test results for each rating dimension. Between subgroups that did and did not meet FA criteria, ratings of high-calorie images showed significant differences in the craving, valence, and arousal dimensions but not in the familiarity dimension. The colors indicate different subgroups, with red denoting the subgroup that met FA criteria and blue denoting the subgroup that did not meet FA criteria [same in panels **(A–C)**]. **(B)** Scatter chart of the Pearson correlation between craving ratings of high-calorie food images and the frequency of cravings in daily life. The positive correlation between craving ratings of high-calorie food images and daily craving experiences (shown in Figure 2B) only appeared in the subgroup that met FA criteria. A linear fit was conducted separately, and the shading within the dotted line indicates the 95% CI. **(C)** Dot plot of the estimated marginal means from the two-way repeated-measures ANOVA with the food subtype as a within-subject factor (six levels) and the FA classification as a between-subject factor (two levels). The greater cravings induced by salty food and sugary drink images (compared to low-calorie food images, shown in Figure 2C) only appeared in the subgroup that met FA criteria. For all subplots, *** indicates *p* < 0.001, ** indicates *p* < 0.01, and “ns” indicates no significance. The error bar indicates the 95% CI.

To test whether the cravings induced by high-calorie food images differed between subgroups of participants that did or did not meet FA criteria, we submitted each rating dimension to an independent sample *T*-test with the FA classification as a group variable. We summarized the results in Figure 3A. Comparing ratings of high-calorie images in the subgroup that met FA criteria to those that did not, all ratings except familiarity showed a significant difference between subgroups [familiarity, *MD* = 0.219, *SE* = 0.260, *t*(211) = 0.843, *p* = 0.400]. The craving [*MD* = 0.885, *SE* = 0.234, *t*(211) = 3.787, *p* < 0.001], valence [*MD* = 0.652, *SE* = 0.208, *t*(211) = 3.137, *p* = 0.002], and arousal ratings [*MD* = 0.792, *SE* = 0.222, *t*(211) = 3.565, *p* < 0.001] were all higher in the subgroup that met FA criteria. We repeated the Pearson correlation analysis and only observed a positive correlation between craving ratings and daily craving experiences in the subgroup that met FA criteria [*r*(122) = 0.270, *p* = 0.003]. In contrast, no significant correlation was found in the subgroup that did not meet FA criteria [*r*(91) = −0.041, *p* = 0.703, Figure 3B]. These results suggested that the positive correlation between craving ratings and daily craving experiences observed in our sample was primarily driven by the subgroup that met FA criteria.

To further examine whether the impact of high-calorie subtypes also arose from the subgroup that met FA criteria, we adopted a two-way repeated-measures ANOVA with food subtype as a within-subject factor (six levels including five high-calorie subtypes and the low-calorie food images as reference) and the FA classification as a between-subject factor. We summarized the results in Figure 3C. The interaction effect of food subtypes and FA classification was significant [*F*(5,1055) = 67.6, *p* = 0.002, η^2^_*p*_ = 0.018], and *post hoc* analysis revealed that in the subgroup that met FA criteria, the images of salty foods [*MD* = 1.617, *SE* = 0.308, *t*(211) = 5.249, *p*_*tukey*_ < 0.001] and sugary drinks [*MD* = 1.094, *SE* = 0.318, *t*(211) = 3.440, *p*_*tukey*_ = 0.033] induced greater cravings than the images of low-calorie foods, however, in those who did not meet FA criteria, none of the high-calorie subtypes induced greater cravings than the low-calorie food images (all *p*_*tukey*_ > 0.05).

## Discussion

We attempted to develop and validate a food picture library, the PLCF, that would be more applicable to investigating high-calorie food preferences in populations with overweight or obesity. To achieve this goal, we defined five subtypes of high-calorie foods with reference to the YFAS 2.0 and collected 100 high-resolution food pictures that were later rated by 241 individuals with overweight or obesity. We observed that the greater cravings induced by high-calorie food images (compared to low-calorie images) were associated only with certain high-calorie subtypes (i.e., salty foods and sugary drinks). Furthermore, we conducted a subgroup analysis of individuals according to whether they did or did not meet FA criteria. We found that the greater cravings induced by certain high-calorie images were driven by the subgroup that met FA criteria.

Consistent with our hypothesis that not all high-calorie food cues have the same effect, the greater cravings associated with high-calorie food images (compared to low-calorie food images) were only present for certain subtypes of food. Salty foods and sugary drinks were the subtypes that evoked greater cravings in our Chinese sample. This result differs from the investigation in a Western cultural context that reported more significant cravings for sugary and fatty foods (Deglaire et al., 2015; Borisenkov et al., 2021). More significant cravings may represent the “addiction risk” of specific foods. Although most studies have focused on the addiction risk of sugary and fatty foods (Rozin et al., 1991; Gearhardt and Schulte, 2021), our results highlighted the potential addiction risk of salty foods, which may act as mild opiate agonists to indirectly regulate dopamine signaling and thereby increase the risk of overeating behaviors (Cocores and Gold, 2009).

We further performed a subgroup analysis to investigate the impact of FA characteristics on the manifestation of food cravings. We found that the associations observed in the whole sample (i.e., the association between high-calorie cravings and daily craving experiences, and the greater cravings induced by salty foods and sugary drinks) only appeared in the subgroup that met FA criteria and were not present in the subgroup that did not. These results are consistent with other studies in which individuals who met FA criteria reported significantly greater subjective cravings than those who did not (Schulte et al., 2019; Schulte and Gearhardt, 2021). Our results further indicated that individuals who meet FA criteria might have a higher risk of overconsuming high-calorie foods and suggests that clinicians should prioritize them for dietary intervention.

These findings are preliminary and should be interpreted carefully with several limitations. First, the food pictures selected for PLCF have non-homogeneous backgrounds without a strict balance of physical parameters, which may distract the raters from the content of the food and affect the ratings. More research is needed to clarify the source of the differences between the subtypes of high-calorie foods observed in this study (i.e., whether the differences are induced primarily by the differences in the content of foods or are also significantly influenced by other physical information in the picture). Second, the BMI used to define participants as overweight or obese in the current study was converted from the participants’ self-reported height and weight, which may limit further quantitative analysis related to BMI. Third, the rating procedure was not conducted in a laboratory setting; therefore, we did not control the fasting time of the raters, which may have increased the variability of craving ratings. Fourth, the design of the PLCF, which was based on nominations of foods by local participants in a specific Chinese cultural context, may undermine its effectiveness in inducing food cravings in residents of other regions and needs to be expanded to include other regional food items. Finally, our findings mainly depend on subjective reports and could be improved by combining such reports with behavioral or physiological indicators in further studies. In addition, our previous research found that even picture-induced cravings carry the property of dynamic change, and dynamic stimulus materials [e.g., animated Graphics Interchange Format (GIF) filess or video clips] can evoke stronger cravings (Gu et al., 2021). Therefore, in the process of expanding the library in the future, manifestations of stimuli could be further enriched with dynamic images to explore the essence of cravings.

## Data availability statement

The datasets presented in this study can be found in online repositories. The names of the repository/repositories and accession number(s) can be found below: https://osf.io/hy5g8/. The original pictures and associated ratings are publicly available on the Open Science Framework website (https://osf.io/hy5g8/).

## Ethics statement

The studies involving human participants were reviewed and approved by the Ethics Committee of Shanghai Sixth People’s Hospital [2020-219-(1)]. The patients/participants provided their written informed consent to participate in this study.

## Author contributions

H-TC and H-WZ: conceptualization and investigation. H-TC and HZ: data curation, methodology, and formal analysis. J-ZD and X-DH: resources. H-TC: writing—original draft preparation and visualization. H-WZ, HZ, and T-FY: writing—review and editing. T-FY, J-ZD, and X-DH: supervision and project administration. H-WZ and T-FY: funding acquisition. All authors read and final approval of the submitted and published versions.

## Footnotes

1
https://osf.io/hy5g8/

2
www.wjx.cn

3
www.r-project.org/

4
www.jamovi.org/
